# ATP6AP1 is a potential prognostic biomarker and is associated with iron metabolism in breast cancer

**DOI:** 10.3389/fgene.2022.958290

**Published:** 2022-09-06

**Authors:** Ye Tian, Ming Gao, Liang Huang, Hu Zhou, Juan Wang

**Affiliations:** ^1^ Department of Thyroid and Breast Surgery, Wuhan No, 1 Hospital, Wuhan, China; ^2^ Department of Blood Transfusion, Tongji Hospital, Tongji Medical College, Huazhong University of Science and Technology, Wuhan, China

**Keywords:** ATP6AP1, breast cancer, iron metabolism, immune infiltration, prognosis

## Abstract

Cancer occurrence and progression may be facilitated by aberrant expression of ATPase H+ transporting accessory protein 1 (ATP6AP1). However, the clinical relevance of ATP6AP1 in breast cancer remains unclear. In this study, we investigated the association between ATP6AP1 and breast cancer. Data collected from patients with breast cancer from the Gene Expression Omnibus (GEO) and The Cancer Genome Atlas (TCGA) were used in this study. To determine the relationship between ATP6AP1 and breast cancer survival rates, Kaplan-Meier analysis was used. To determine the prognostic value of ATP6AP1, a receiver operating characteristic (ROC) curve was constructed. To identify the major pathways involving ATP6AP1, we performed functional enrichment analysis using gene set enrichment analysis (GSEA). We analyzed the association between ATP6AP1 expression and tumor immunity using the ESTIMATE algorithm and single-sample GSEA (ssGSEA). A nomogram based on a Cox regression analysis was constructed to predict the impact of ATP6AP1 on prognosis. ATP6AP1 expression was significantly upregulated in breast cancer tissues. Moreover, patients with elevated ATP6AP1 expression had shorter total survival rates than those with lower expression levels (*p* = 0.032). The area under the receiver operating characteristic curve for ATP6AP1 was 0.939. Gene set enrichment analysis revealed that reaction iron uptake and transport, proteasome degradation, glutathione metabolism, and pyruvate metabolism were enriched in the ATP6AP1 high expression phenotype. The relationship between immune infiltration cells and ATP6AP1 expression, including macrophages, B cells, dendritic cells, cytotoxic cells, NK cells, and T cells, was found to be negative, suggesting that ATP6AP1 overexpression results in immunosuppression. Based on the Cox regression analyses, the calibration plot of the nomogram demonstrated effective performance in predicting breast cancer patients. ATP6AP1 may facilitate breast cancer progression by inhibiting antitumor immunity and promoting iron metabolism and may be a biomarker for breast cancer prognosis.

## Introduction

Breast cancer is the most common type of cancer in women and one of the main causes of cancer-related deaths globally (Sung et al., 2021). Despite the advances in diagnostics and treatments, 20%–30% of patients with primary breast cancer experience recurrence and distant metastasis (Duma, 2018). Furthermore, advanced breast cancer has a poor prognosis (Wang et al., 2017). Although research on breast cancer pathogenesis has made great strides, including the tumor microenvironment (TME) and energy metabolism, the underlying pathogenesis of breast cancer must be clarified (Zhou et al., 2019). Cancer antigen 15-3 (CA15-3) is used as a biomarker for relapse and therapeutic efficacy in patients with breast cancer (Liu et al., 2021). However, the reliability of this method is unsatisfactory (Talaat et al., 2020). Hence, it is imperative to screen novel biomarkers to predict prognosis, monitor metastasis, identify therapeutic targets, and investigate the potential mechanisms of breast cancer.

Iron is a vital element in life because it is involved in many metabolic processes (Song and Dunaief, 2013). Among its many functions, iron is required for energy metabolism, oxygen transport and storage, antioxidant activity, and DNA synthesis (Schmelz et al., 2009). Tumor cells modify their iron metabolism to maximize absorption and minimize outflow, increasing labile iron (Duan et al., 2019). The abnormal metabolism and proliferation of cancer cells require a high level of iron and, consequently, tumor tissues contain a higher amount of iron than normal tissues (Xu et al., 2020). There is also evidence that an abnormal iron metabolism may lead to tumor initiation, proliferation, and metastasis (Raggi et al., 2017). Chelators for iron, originally designed for treating iron overload, can also prevent tumor progression (Chen et al., 2019). However, there is little information on iron metabolism-associated genes and their clinical relevance in malignant tumors (Miller et al., 2011). Therefore, screening for iron metabolism-associated genes that are closely related to tumorigenesis and tumor progression is important for clinical applications.

ATPase H+ -transporting accessory protein 1 (ATP6AP1) is a member of the V-ATPase complex that functions as an accessory subunit and is related to the V-ATPase membrane domain (V0) (Yang et al., 2012). V-ATPase is believed to be mainly involved in tumor growth and metastasis *via* its ability to increase H+ secretion, resulting in tumor cell survival in hypoxia and an acidic tumor microenvironment (Collins and Forgac, 2020). According to a recent study, salivary autoantibodies against ATP6AP1 may serve as biomarkers for the early detection of breast cancer (Arif et al., 2015). Notably, recent research has indicated that ATP6AP1 is an iron metabolism-associated gene (Zhang et al., 2020). Nevertheless, ATP6AP1’s underlying activities and mechanisms in tumor growth and immunology remain partially characterized. To date, there are few studies on the relationship between ATP6AP1 and breast cancer (Arif et al., 2015; Wang et al., 2021).

Therefore, we aimed to establish a link between ATP6AP1 and breast cancer and investigate its prognostic value. To accomplish this goal, bioinformatics was used to analyze public datasets from TCGA and GEO. To clarify its function in breast cancer, we identified the genes and pathways related to ATP6AP1. Furthermore, we examined the association between tumor immune infiltration and ATP6AP1 expression in breast cancer. We developed a nomogram to aid in the prediction of breast cancer patient prognosis, which included ATP6AP1 expression levels and clinicopathological parameters that were significant in Cox regression analyses. Based on these findings, ATP6AP1 may be used as a biomarker for the prognosis and diagnosis of breast cancer.

## Materials and methods

### Source data

A total of 1,109 cases with RNA-seq data (HTSeq-FPKM and HTSeq-counts) and clinical information from TCGA-BRCA were obtained from the Genomic Data Commons (GDC) TCGA data portal (https://portal.gdc.cancer.gov/), and 112 paired tumor tissue and adjacent normal tissue samples were included in these data. The GEO database was used to download the GSE45827 (Gruosso et al., 2016) and GSE42568 (Clarke et al., 2013) datasets, and the platform used for both datasets was GPL570 [HG-U133_Plus_2] Affymetrix Human Genome U133 Plus 2.0 Array. The GSE45827 dataset consisted of 11 normal samples and 130 breast cancer samples, whereas the GSE42568 dataset contained 17 normal samples and 104 breast cancer samples. Iron metabolism-associated genes were identified in Zhang’s research (Zhang et al., 2020), including 70 genes, such as *NUBP1*, *FBXL5*, *TMEM199*, *HJV*, *ALAS1*, *ALAS2*, and *ISCU.* Informed consent and ethics approval was not required, as all data were obtained from TCGA and GEO.

### Co-expression analysis

The LinkedOmics database is an online resource that uses multi-omics to analyze data on 32 cancer types (http://www.linkedomics.org/). The database contains three analytical modules: LinkFinder, LinkInterpreter, and LinkCompare (Vasaikar et al., 2018). Volcano plots and heat maps were used to display the genes co-expressed with ATP6AP1 in breast cancer, and Spearman correlation tests were used to test correlations.

### Functional enrichment analysis

Functional enrichment analyses, comprising gene ontology (GO) analysis consisting of cellular component (CC), biological process (BP), molecular function (MF), and Kyoto Encyclopedia of Genes and Genomes (KEGG) pathway analysis, were performed using the clusterProfiler (Yu et al., 2012) package in R with |R| > 0.4 and FDR < 0.05. A *p*-value lower than 0.05 was considered statistically significant. We performed GSEA to determine if previously described functional or pathway groups of genes significantly differed between those that are negatively and positively linked with ATP6AP1. The “c2. cp.v7.2. symbols.gmt [Curated]” gene set was downloaded from the MSigDB database for GSEA analysis; a *p*-value < 0.05 was considered to be significantly enriched. In addition, using the R package GSVA (Hänzelmann et al., 2013), the scores of related pathways were calculated according to the gene expression matrix of each sample by ssGSEA, and the enrichment functions were differentially screened using the R package limma (Ritchie et al., 2015).

### Construction of interaction networks

The Search Tool for the Retrieval of Interacting Genes (STRING) database is a global resource for predicting protein-protein interaction networks (Szklarczyk et al., 2021). We generated a protein-protein interaction (PPI) network of genes to explore the interactions among genes, including ATP6AP1, using the STRING database. The starBase database is an invaluable resource for studying non-coding RNAs such as miRNAs, circRNAs, and lncRNAs (Li et al., 2014). Potential miRNAs binding to ATP6AP1 were predicted using the starBase database, and the parameters were set as Genome (human), Clade (mammal), CLIP-Data (≥ 1), Assembly (hg19), Degradome-Data (≥ 1), pan-Cancer (≥ 1), program Num (≥ 1), and program (None).

The PROMO database was used to predict transcription factor binding sites (TFBS) (Messeguer et al., 2002; Farre et al., 2003). Using the PROMO database, we predicted the transcription factors binding to ATP6AP1, in which the maximum matrix dissimilarity rate was set to 5%. The Comparative Toxicogenomics Database (CTD) is an open resource that contains links between chemicals and diseases (Davis et al., 2021). We predicted the drugs associated with ATP6AP1 using this database. We constructed interaction networks using the R package igraph (Csardi and Nepusz, 2006).

### Immune infiltration analysis

Immune infiltration of breast cancer was analyzed using the ssGSEA method with R package GSVA for a total of 24 distinct immune cell subtypes in breast cancer samples. Based on Spearman’s correlation, the link between ATP6AP1 and these immune cells was investigated. We used the R package ESTIMATE (Yoshihara et al., 2013) to evaluate the ratio of the immune-stromal component in the TME for each breast cancer sample. The displayed scores consisted of the StromalScore, ImmuneScore, and ESTIMATEScore. Three types of scores were shown to be highly related to the stromal/immune ratio as well as the sum of the two.

### Clinical correlation and prognosis analysis

To appraise the efficiency of ATP6AP1 expression in discriminating breast cancer from healthy samples, ROC analysis was conducted. To assess ATP6AP1’s predictive significance in breast cancer, the Kaplan-Meier method was used. To assess the impact of prognostic factors in patients with breast cancer, Cox regression analysis was performed. Based on the Cox regression analysis findings, a nomogram was constructed to predict the survival probability at 2 years, 4 years, and 6 years. A nomogram including significant clinical characteristics and a calibration plot were generated using the R package rms (Eng et al., 2015). To evaluate the accuracy of the nomogram forecast based on the prognostic model, a calibration plot was constructed. In addition, data from the TCGA were randomly divided into training and testing sets, and the nomogram was assessed using the ROC curves and decision curve analysis (DCA) curves, respectively.

### Statistical analysis

Statistical significance for normally distributed variables was determined using Student’s *t*-tests, and we employed the Wilcoxon rank-sum test (also known as the Mann–Whitney *U*-test) for data that were not typically distributed. The chi-square test or Fisher’s exact test was used to compare categorical variables between the two groups. *p*-values were always bilateral, and *p*-values less than 0.05 were deemed statistically significant.

## Results

### Differential gene expression analysis and clinical value of ATP6AP1 in breast cancer

**FIGURE 1:**
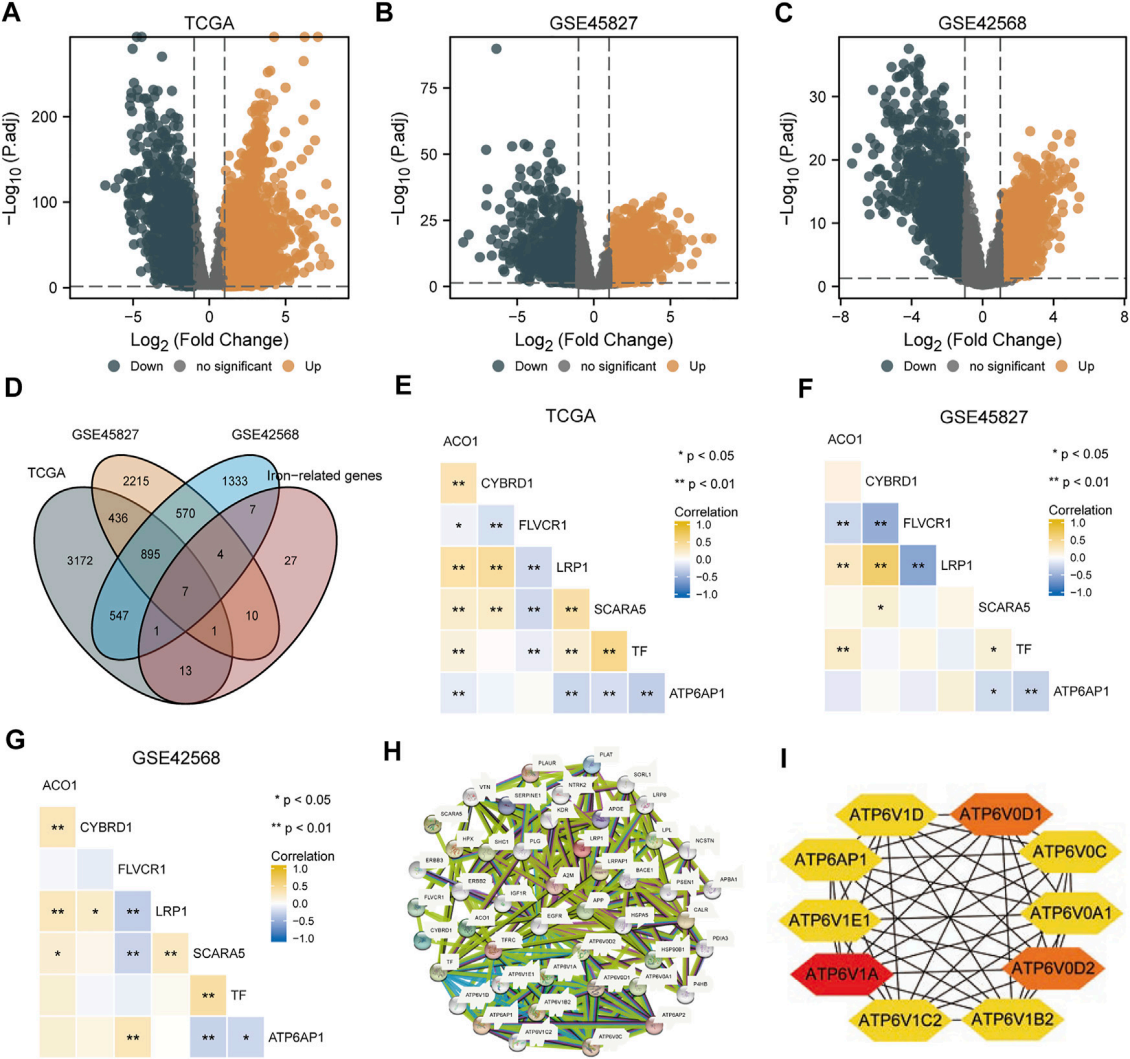
DEG analyses of three datasets. **(A)** TCGA-BRCA dataset’s volcano map shows differentially expressed genes. **(B)** The GSE45827 dataset shows a volcano map of genes with differential expression. **(C)** The GSE42568 dataset shows a volcano map of differentially expressed genes. **(D)** Venn diagram of differential expressed genes in three datasets and iron metabolism-associated genes. **(E–G)** Heat maps of correlation among seven iron metabolism-related genes in the TCGA-BRCA, GSE45827, and GSE42568 datasets. **(H)** A PPI network was constructed by differentially expressed genes and iron metabolism-associated genes. **(I)** Top 10 hub genes extracted by CytoHubba.

The TCGA-BRCA, GSE45827, and GSE42568 datasets were analyzed using R package limma to identify differentially expressed genes (DEGs), and 5,072, 4,138, and 3,364 DEGs were identified, respectively. The results are shown in the volcano plots in Figures 1A–C, respectively. Taking the intersection of the DEGs in the three datasets and iron metabolism-related genes, we identified seven genes: *FLVCR1*, *ATP6AP1*, *CYBRD1*, *LRP1*, *ACO1*, *TF*, and *SCARA5* (Figure 1D). Heat maps of correlation among seven iron metabolism-related genes in the TCGA-BRCA, GSE45827, and GSE42568 datasets were shown in Figures 1E–G respectively. Among them, ATP6AP1 was significantly negatively correlated with TF and SCARA5, TF was significantly positively correlated with SCARA5, FLVCR1 was significantly negatively correlated with LRP1, and CYBRD1 was significantly positively correlated with LRP1 (*p* < 0.05). A PPI network of ATP6AP1 and its potential co-expression genes was constructed using the STRING database (Figure 1H). In addition, CytoHubba, a Cytoscape plug-in, was applied to detect hub genes, and the proper order of the top 10 hub genes was *ATP6V1A*, *ATP6V0D2*, *ATP6V0D1*, *ATP6AP1*, *ATP6V1D*, *ATP6V1E1*, *ATP6V0A1*, *ATP6V1C2*, *ATP6V1B2*, and *ATP6V0C* (Figure 1I).

**FIGURE 2:**
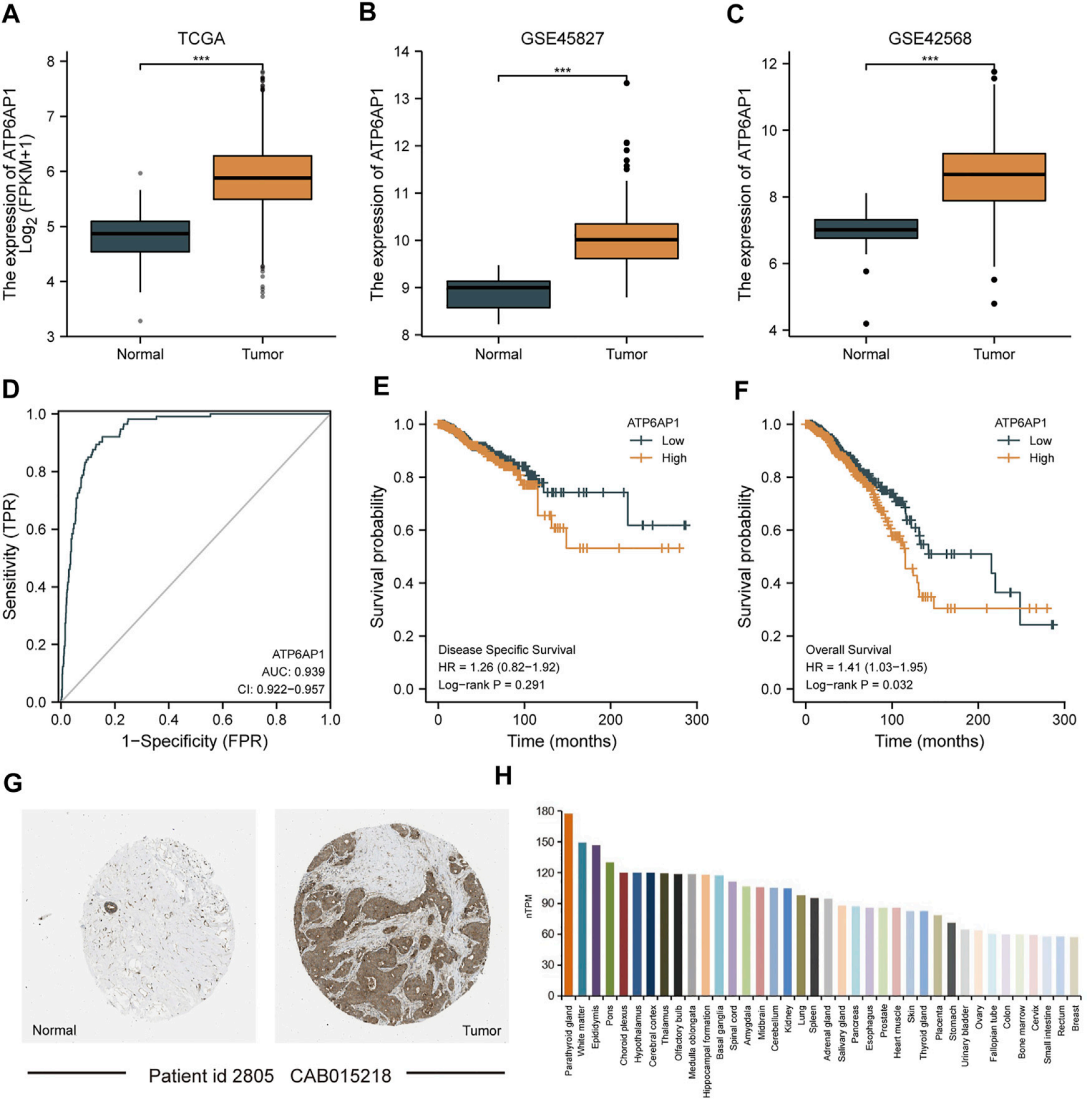
ATP6AP1 expression in patients with breast cancer. **(A)** Expression of ATP6AP1 in unpaired samples in the TCGA-BRCA dataset. **(B)** Expression of ATP6AP1 in the GSE45827 dataset. **(C)** Expression of ATP6AP1 in the GSE42568 dataset. **(D)** Evaluation of ATP6AP1 diagnostic efficiency in breast cancer by drawing ROC curve. **(E)** Survival analysis of DSS in the ATP6AP1 group with a greater level of expression and a group with a lower level of expression. **(F)** Survival analysis of OS in ATP6AP1 group with a higher expression level and a group with a lower expression level. **(G)** Protein expression level of ATP6AP1 in breast cancer tissues and in normal breast tissues. **(H)** Tissue distribution of top 38 in the expression of ATP6AP1 in systemic tissues. ns, non-significant (*p* ≥ 0.05); *p < 0.05; **p < 0.01; ***p < 0.001.

As shown in Figure 2, in the TCGA-BRCA, GSE45827, and GSE42568 datasets, ATP6AP1 expression was substantially lower in normal tissues than in breast cancer tissues (*p* < 0.001). The area under the curve (AUC) of ATP6AP1 expression for distinguishing tumors from normal tissues was 0.939 on the ROC curve, indicating that ATP6AP1 might be a potential diagnostic marker for breast cancer. High ATP6AP1 expression was associated with a worse prognosis (*p* = 0.032), as revealed by the Kaplan-Meier survival analysis. Based on data from the Human Protein Atlas (HPA) database, the level of ATP6AP1 protein expression in breast cancer tissues was significantly higher than in normal breast tissues (Figure 2G). Furthermore, Figure 2H shows the top 38 tissues of whole-body tissues in terms of ATP6AP1 expression.

### Co-expression analysis of ATP6AP1 in breast cancer

**FIGURE 3:**
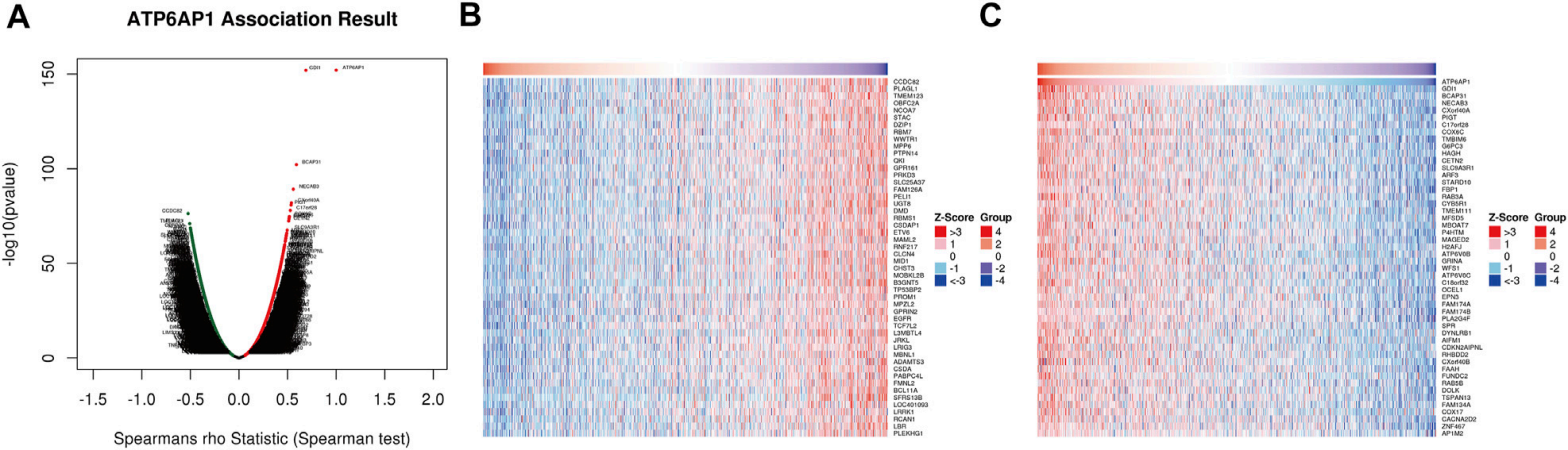
Co-expressed genes related to ATP6AP1. **(A)** Analysis of Spearman correlation found that co-expressed genes were strongly related to ATP6AP1 in breast cancer; red and green dots represent positively and negatively correlated genes, respectively. **(B)** ATP6AP1 was negatively correlated with the first 50 genes in the heat map. **(C)** ATP6AP1 was positively linked with the first 50 genes in the heat map.

The functional modules of LinkedOmics were used to detect genes that were co-expressed with ATP6AP1 in breast cancer to further understand ATP6AP1’s biological functions in the disease. A total of 5,448 genes were found to be significantly positively associated with ATP6AP1, and ATP6AP1 was significantly negatively associated with 8,650 genes (FDR <0.05; Figure 3A). According to the heat maps, the top 50 genes were found to have a substantial positive correlation with ATP6AP, and the top 50 genes exhibited a significant negative correlation with ATP6AP1 (Figures 3B, C).

### Functional enrichment analyses of ATP6AP1 co-expressed genes in breast cancer

**FIGURE 4:**
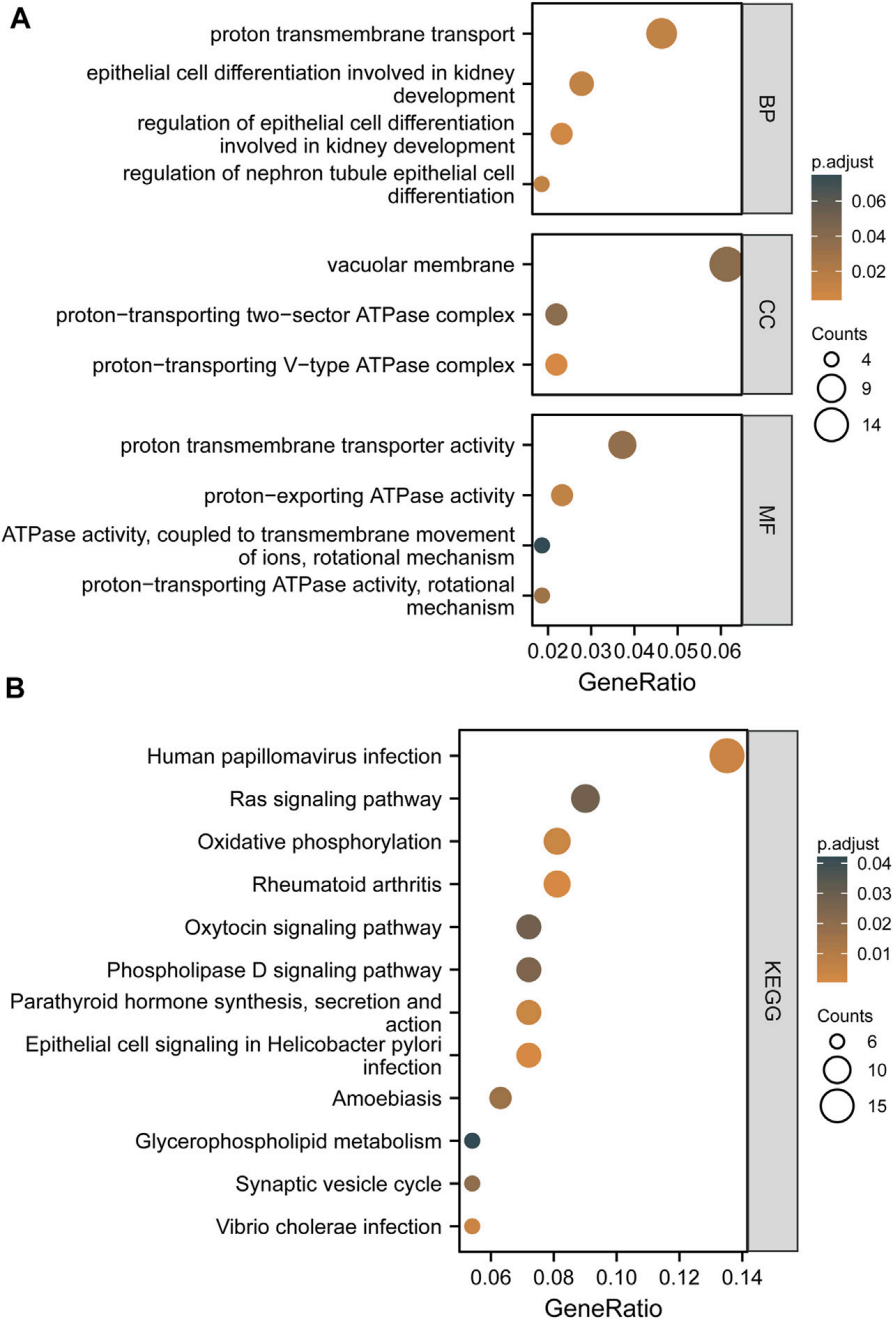
Based on TCGA and GEO, the findings of GO terms and KEGG pathway enrichment of co-expressed genes between high and low ATP6AP1 expression are shown. **(A)** Results of GO enrichment analysis. **(B)** Results of the KEGG pathway enrichment analysis.

According to the threshold criteria (|R| > 0.4 and FDR < 0.05), 259 co-expressed genes highly correlated with ATP6AP1 were identified: 156 genes were negatively correlated, and 103 genes were significantly positively correlated. To further understand the impact of ATP6AP1 in breast cancer, we used GO and KEGG functional enrichment analyses to evaluate co-expressed genes. The GO enrichment results are presented in Figure 4A and Supplementary Table S1. Five enriched GO terms were identified in the biological process classification. These genes were enriched in epithelial cell differentiation involved in kidney development, regulation of epithelial cell differentiation involved in kidney development, nephron tubule epithelial cell differentiation, proton transmembrane transport, and regulation of nephron tubule epithelial cell differentiation. Based on the categorization by “cellular component,” three enriched GO terms were identified, which were related to the vacuolar membrane, proton-transporting two-sector ATPase complex, and proton-transporting V-type ATPase complex. Furthermore, the molecular function category revealed five enriched GO terms significantly associated with proton-transporting ATPase activity, rotational mechanism, proton-exporting ATPase activity, proton transmembrane transporter activity, lipase activity, ATPase activity coupled to transmembrane movement of ions, and rotational mechanism. The results of the KEGG enrichment analysis revealed that co-expressed genes were mostly related to oxytocin signaling pathway, rheumatoid arthritis, epithelial cell signaling in *Helicobacter pylori* infection, human papillomavirus infection, Vibrio cholerae infection, parathyroid hormone synthesis, secretion and action, and amoebiasis (Figure 4B; Supplementary Table S2).

### ATP6AP1-related signaling pathways based on gene set enrichment analysis

**FIGURE 5:**
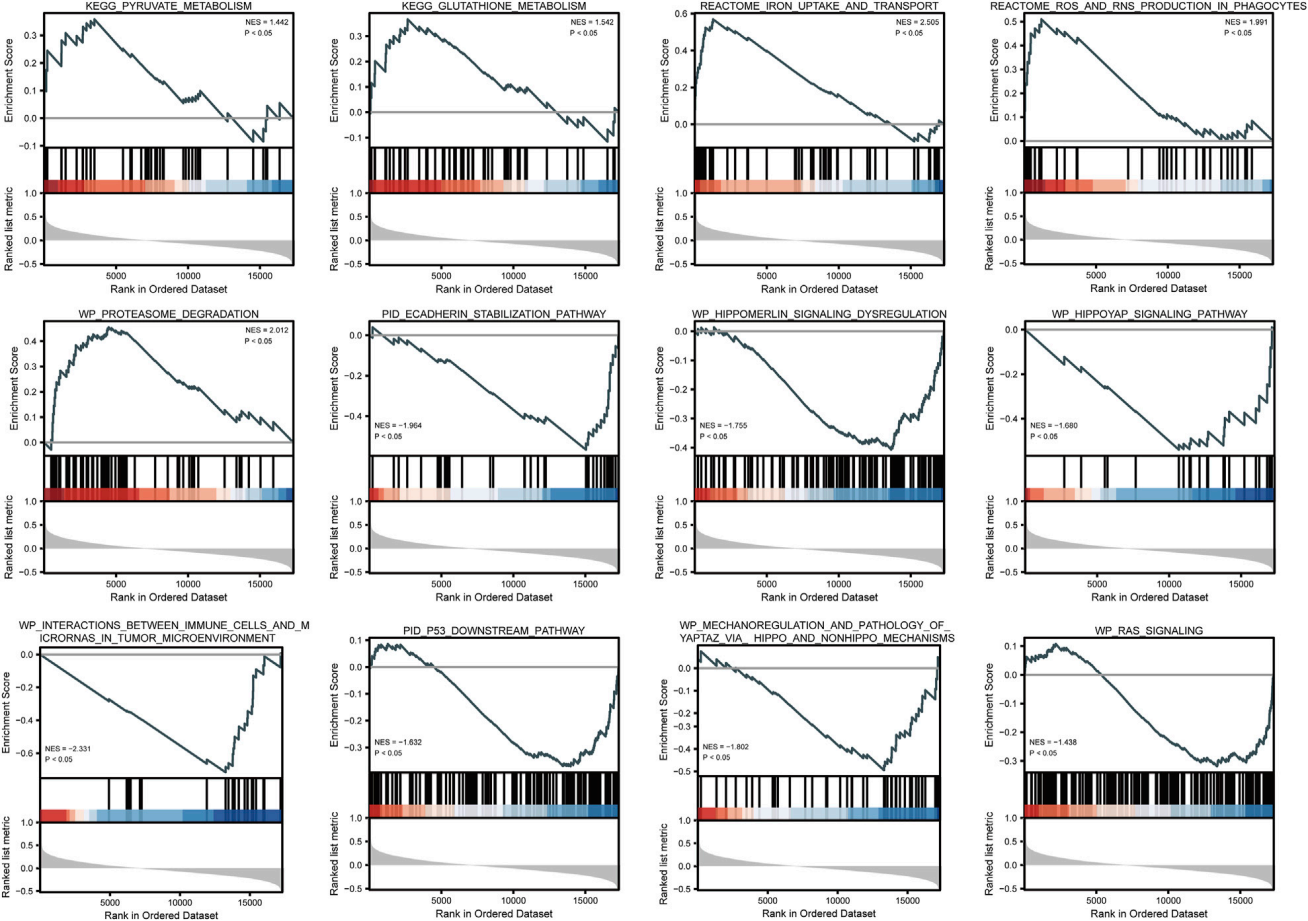
GSEA enrichment plots show several pathways were differentially enriched in ATP6AP1-related breast cancer.

Based on GSEA, five pathways, namely, iron uptake and transport, proteasome degradation, glutathione metabolism, and pyruvate metabolism, were significantly enriched in positively correlated co-expressed genes, whereas seven pathways, including the RAS signaling, P53 downstream, Hippo-YAP signaling, and Hippo-Merlin signaling dysregulation pathways, as well as the mechanoregulation and pathology of YAP/TAZ *via* Hippo and non-Hippo mechanisms, and the E-cadherin stabilization pathways, were significantly enriched in negatively correlated co-expressed genes (Figure 5 and Supplementary Table S3).

**FIGURE 6:**
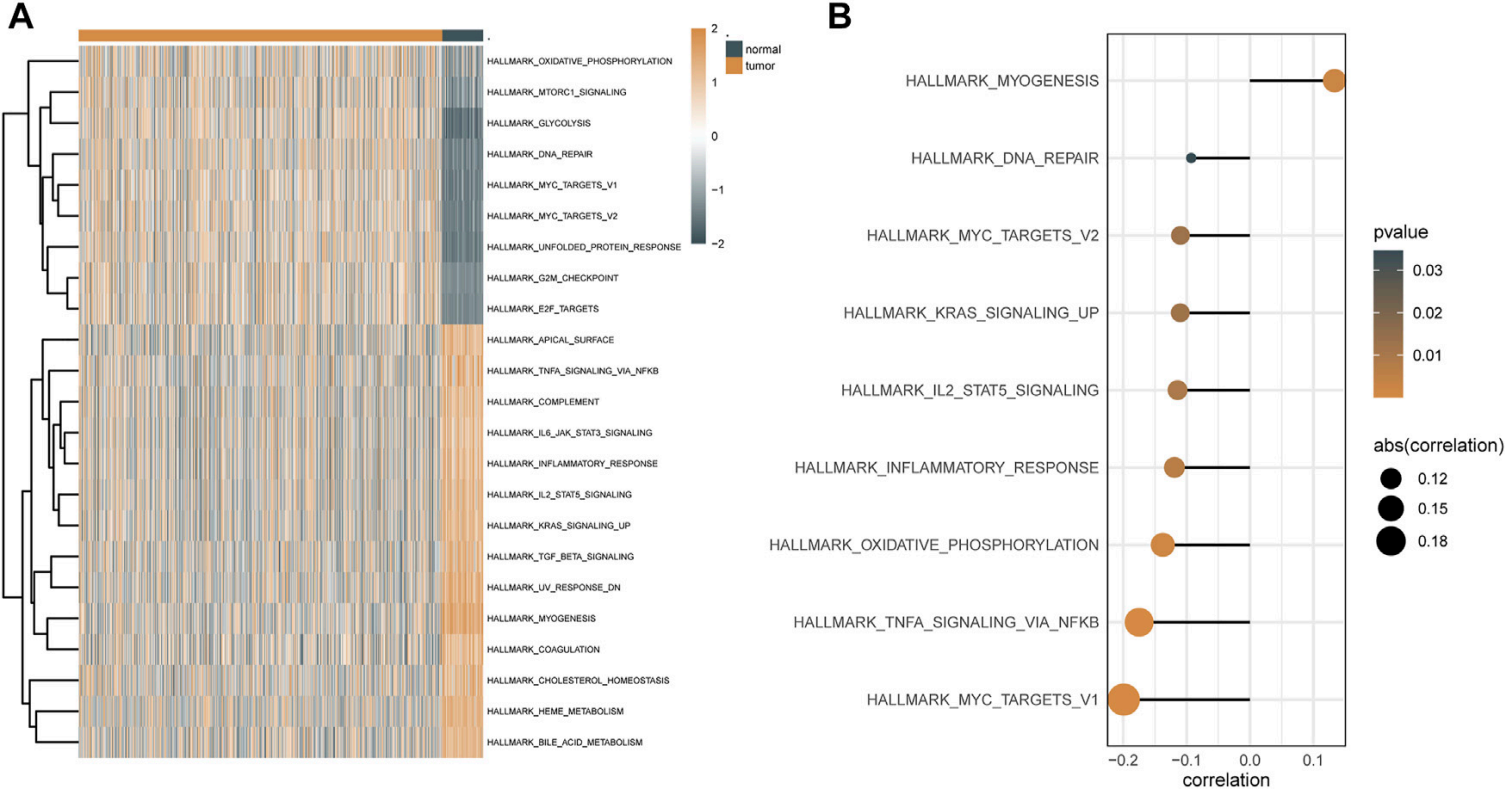
GSVA results based on hallmark gene sets. **(A)** Heat map of differential pathways of GSVA analysis depended mainly on hallmark gene sets. **(B)** Correlation analysis between differential pathways and ATP6AP1 expression.

GSVA was used to examine the dynamics of biological pathways and processes based on hallmark gene sets. Regarding the results of GSVA, the scores of pathways, including DNA repair, glycolysis, and mTORC1 signaling, were higher in malignant tissues than in normal tissues, whereas the scores of pathways, including myogenesis, coagulation, and KRAS, were lower in malignant tissues than in normal tissues (Figure 6A). In addition, based on the correlation analysis between these pathways and the expression level of ATP6AP1, it was discovered that ATP6AP1 expression was adversely linked with the majority of these pathways but only positively correlated with the myogenesis pathway (Figure 6B).

### Construction of ATP6AP1-associated interaction networks in breast cancer

The STRING database was used to build a PPI network of ATP6AP1 and its potential co-expression genes (Supplementary Figure S1A). Based on the regulation of ATP6AP1 by miRNAs, we constructed the target relationship network using the starBase database (Supplementary Figure S1B). The interaction network was constructed based on transcription factors binding to ATP6AP1, as predicted by the PROMO database (Supplementary Figure S1C). The drug-gene interaction network, including ATP6AP1 and related chemical drugs, was constructed based on the CTD (Supplementary Figure S1D).

### Correlation between ATP6AP1 expression and immune infiltration

**FIGURE 7:**
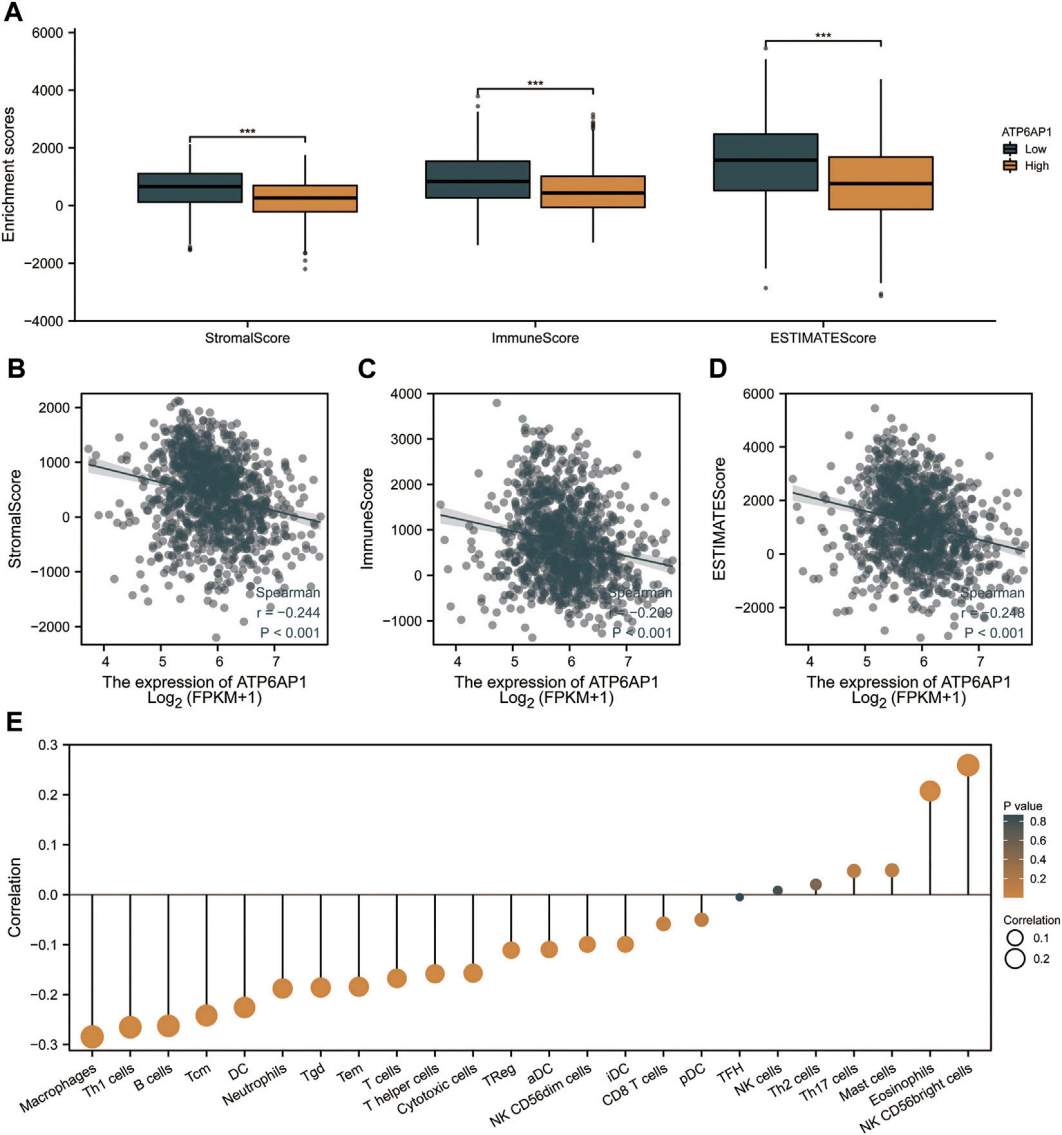
ATP6AP1 expression is associated with immune infiltration in TME. **(A)** Patients with high expression of ATP6AP1 had significantly lower StromalScore, Immune Score, and ESTIMATE Score (*p* < 0.001) in comparison with patients with low expression of ATP6AP1. **(B–D)** StromalScore, ImmuneScore, and ESTIMATES Score were negatively linked with the expression of ATP6AP1 (*p* < 0.001). **(E)** Lollipop plot illustrates the connection between the relative abundance of 24 immune cells and ATP6AP1 expression. ns, non-significant (*p* ≥ 0.05); *p < 0.05; **p < 0.01; ***p < 0.001.

We further analyzed the effect of ATP6AP1 expression on the immunological characteristics of breast cancer patients in TCGA-BRCA. StromalScore, ImmuneScore, and ESTIMATEScore were significantly lower in “high ATP6AP1 expression” breast cancer patients relative to “low ATP6AP1 expression” breast cancer patients (*p* < 0.001; Figure 7A). StromalScore, ImmuneScore, and ESTIMATEScore negatively correlated with the expression of ATP6AP1 (*p* < 0.001; Figures 7B–D). Using ssGSEA, we examined the relationships between immune cell enrichment and ATP6AP1 expression and found that ATP6AP1 expression was positively related to the abundance of eosinophils and CD56bright NK cells, and negatively associated with the abundance of macrophages, Th1 cells, B cells, central memory CD4^+^ T-cells, DC, immature DCs, CD56dim NK cells, cytotoxic cells, NK cells, neutrophils, T-cells, T helper cells, effector memory T-cells, Tgd, Th1 cells, and Tregs (*p* < 0.05; Figure 7E).

### Clinical relevance of ATP6AP1 expression and development of a nomogram based on ATP6AP1

**FIGURE 8:**
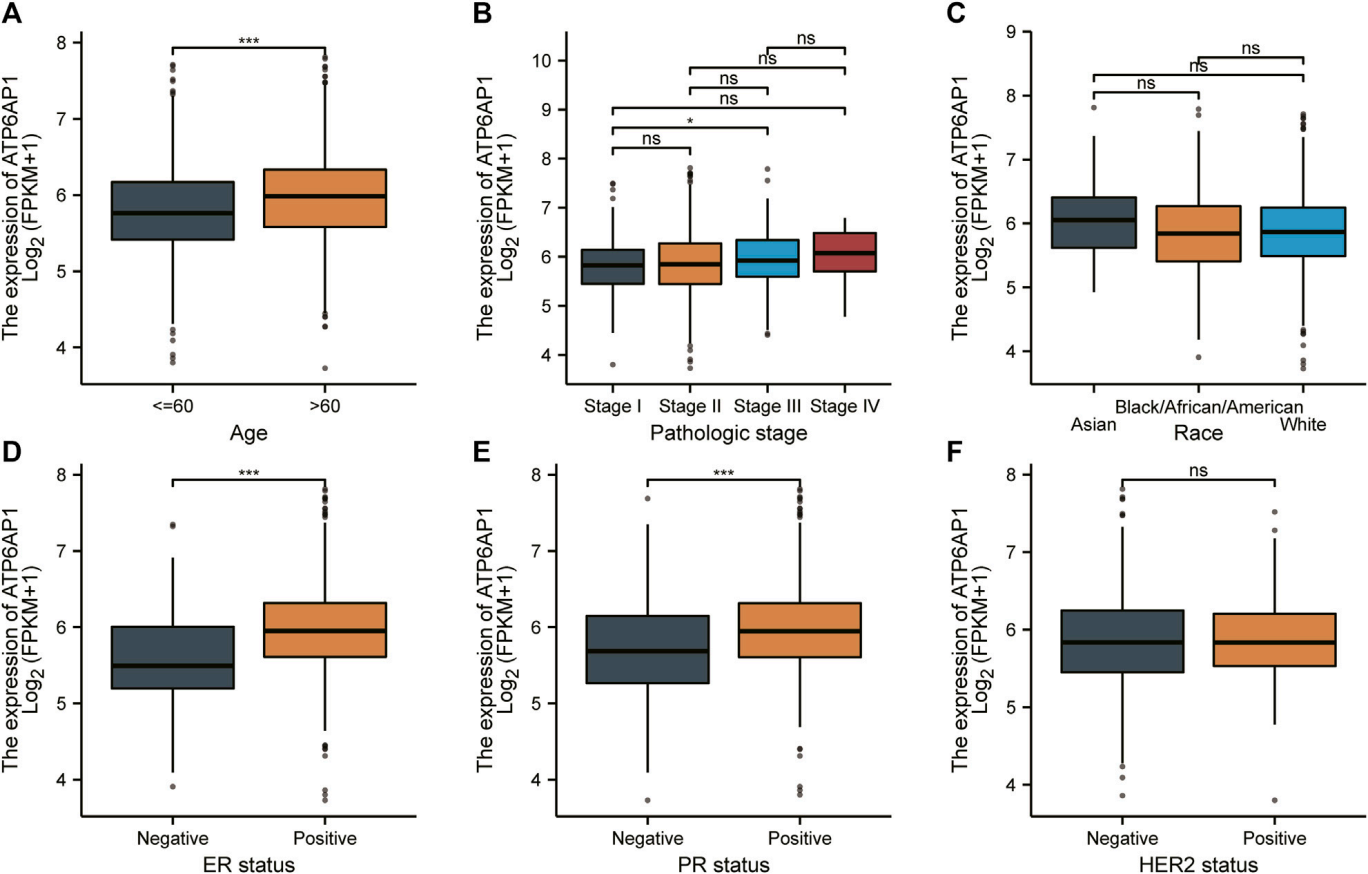
Relationship between ATP6AP1 expression and clinicopathologic characteristics in patients with breast cancer. **(A)** Age. **(B)** Pathologic stage. **(C)** Race. **(D)** ER status. **(E)** PR status. **(F)** HER2 status. ns, non-significant (*p* ≥ 0.05); *p < 0.05; **p < 0.01; ***p < 0.001.

**FIGURE 9:**
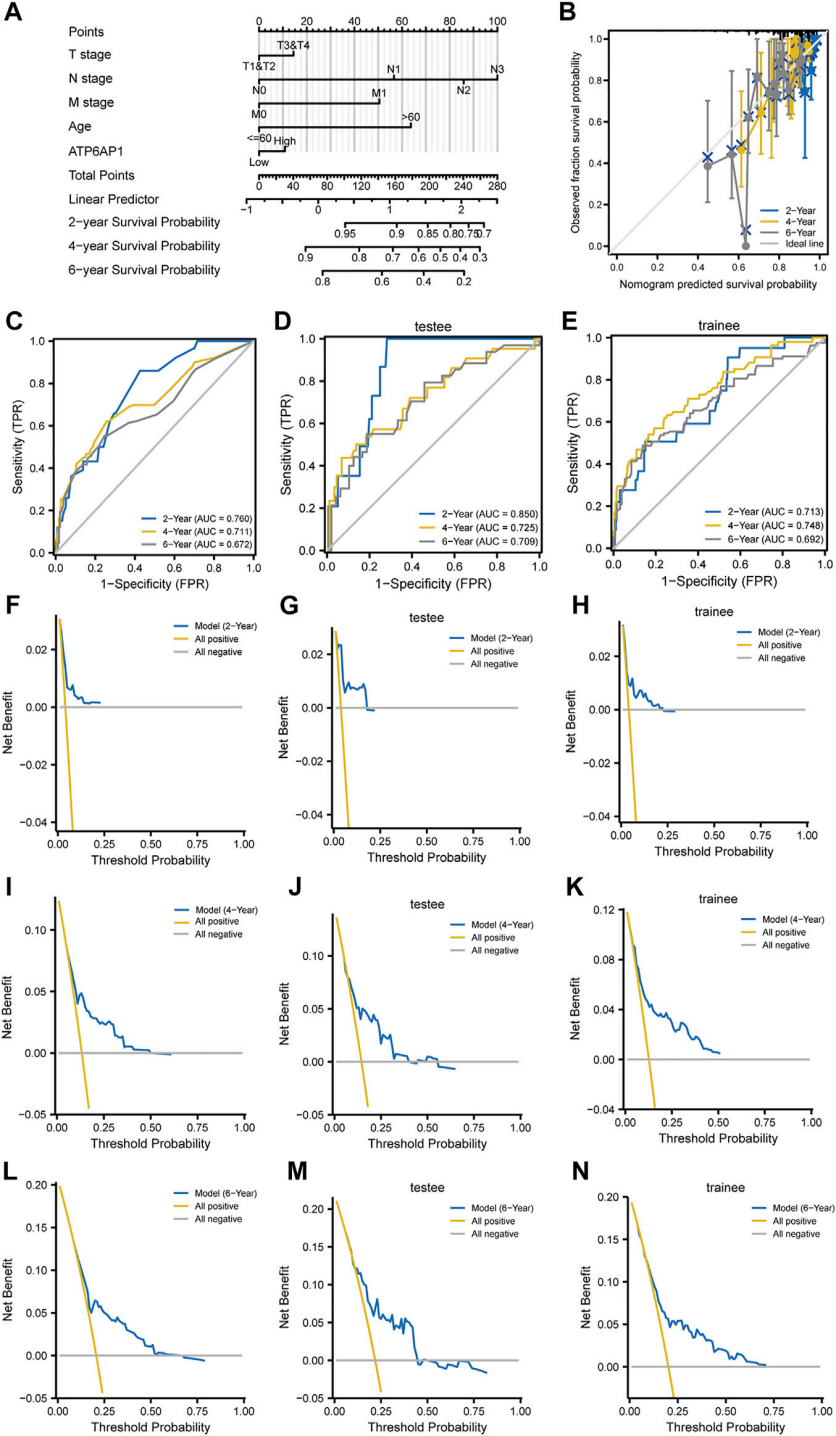
Construction of a prognosis model. **(A)** Breast cancer patients’ 2-, 4-, and 6-year survival probabilities were predicted using a nomogram. **(B)** A nomogram calibration plot for forecasting survival probability at two, four, and 6 years. **(C–E)** ROC curve based on the nomogram prognostic model and ROC curves based on trainee and testee groups. **(F–N)** DCA curves based on the nomogram prognostic model and DCA curves are based on trainee and testee groups.

To investigate the connection between ATP6AP1 expression and clinicopathological parameters of patients in the TCGA-BRCA cohort, based on the median value, we separated breast cancer samples into two groups: high expression and low expression (Supplementary Table S4). Therefore, the results indicate that ATP6AP1 was significantly correlated with age, ER status, and PR status, all with *p*-values <0.001 (Figures 8A,D,E). The expression of ATP6AP1 was not associated with pathologic stage, race, and HER2 status (Figures 8B,C,F). Univariate Cox regression analysis revealed an association between ATP6AP1 and breast cancer prognosis. Univariate and multivariate Cox regression analyses revealed that the patient’s age (*p* < 0.001) was an independent prognostic factor in the TCGA-BRCA cohort (Supplementary Table S5). Previously, age has been reported to be a prognostic factor for breast cancer (Garcia-Estevez et al., 2021), and metastatic breast cancer patients’ age at diagnosis was an independent prognostic factor (Chen et al., 2017). A nomogram based on ATP6AP1 and other clinicopathological parameters was constructed to help clinicians predict the prognosis of patients with breast cancer (Figure 9A) (Gittleman et al., 2020). The calibration plot of the nomogram (Figure 9B), ROC curves (Figures 9C–E), and DCA curves (Figures 9F–N) suggest that the model demonstrated a strong predictive value for breast cancer patient prognosis at 2-, 4-, and 6 years.

## Discussion

ATP6AP1 is a vacuolar (V)-ATPase proton pump accessory subunit required for luminal acidification of secretory vesicles, Golgi, and lysosomes (Kanaki et al., 2019). The ATP6AP1 protein is expressed in numerous tumors, including head and neck carcinoma, lung cancer, and leukemia (Arif et al., 2015). To survive in a hypoxic microenvironment, tumor cells utilize this ATPase system to maintain an acidic pH (Hernandez et al., 2012). Although V-ATPases have been proven to be carcinogenic in certain neoplasms, the prognostic value and underlying mechanism of ATP6AP1 in breast cancer need to be fully characterized. Our results suggest that the ATP6AP1 levels in breast cancer tissues were substantially higher than those in normal breast tissues. Based on the ROC analysis, the AUC was 0.939, suggesting that ATP6AP1 expression could have potential diagnostic value in distinguishing breast cancer from normal tissues. Moreover, the Kaplan-Meier method shows that ATP6AP1 expression levels in patients with breast cancer might predict overall survival, with higher levels suggesting a worse result. Therefore, ATP6AP1 may be used as a biomarker for the diagnosis and prognosis of breast cancer.

The association between immune cell infiltration and ATP6AP1 expression levels was assessed by ssGSEA using Spearman correlation. Previous research has demonstrated the relationship between ATP6AP1 and various immune cells resulting in a change in immunological environment may contribute to the poor outcomes (Wang et al., 2021). Our results show that ATP6AP1 expression was inversely associated with macrophages, Th1 cells, B cells, central memory CD4^+^ T-cells, DC, immature DCs, neutrophils, cytotoxic cells, CD56dim NK cells, NK cells, T helper cells, T-cells, effector memory T-cells, Tgd, Th1 cells, and Tregs but positively associated with eosinophils and CD56bright NK cells. M1 macrophages can destroy tumor cells, whereas M2 macrophages act as tumorigenic macrophages that facilitate tumor initiation, metastasis, and progression (Yuan et al., 2020). Several studies have demonstrated that DCs infiltrate tumors, impact the TME, and initiate immunity against cancer cells (Zhang et al., 2018). Tumor cells can be killed by innate NK cells (Filtjens et al., 2016). It has been shown that eosinophils play a role in angiogenesis and tumor metastasis and that greater numbers of eosinophils are associated with a poor prognosis (Kang et al., 2021). Thus, ATP6AP1 overexpression in breast cancer tissues may inhibit cytotoxic activity against tumor cells, leading to a poor prognosis based on immune infiltration analysis. The TME is mainly composed of recruited immune and resident stromal cells. ATP6AP1 expression was negatively correlated with the StromalScore, ImmuneScore, and ESTIMATEScore. In our study, we demonstrated that ATP6AP1 may play an important role in immunological suppression and may lead to worse outcomes in patients with breast cancer through TME regulation. Furthermore, we suggest that patients with breast cancer who have high ATP6AP1 expression may not respond well to immunotherapy.

Detecting the genes co-expressed with ATP6AP1 helped us gain a better understanding of ATP6AP1’s biological activities in breast cancer. Among the genes that exhibited a significant negative correlation with ATP6AP1, EGFR was found to be co-expressed with ATP6AP1. Aberrant EGFR is associated with aggressive clinical behavior in breast cancer; moreover, elevated EGFR levels increase the likelihood of developing ER-positive breast cancer (Li et al., 2015). *ATP6V0C* was found to be co-expressed with *ATP6AP1* among the genes that showed a significant positive correlation. *ATP6V0C* encodes a component of an ATP-driven proton pump V-ATPase that regulates autolysosome production and the acidic TME (Kim et al., 2018). In addition, KEGG and GO analyses were performed for the co-expressed genes. These results show a link between *ATP6AP1* expression and proton transmembrane transport. These co-expression genes are closely associated with ATPase activity. Further research is required to elucidate the underlying regulatory mechanisms.

Using GSEA, we discovered that the co-expression genes that were positively connected with ATP6AP1 participate in the response to iron absorption and transport, proteasome degradation, glutathione metabolism, and pyruvate metabolism. Therefore, high ATP6AP1 expression was closely related to iron uptake and transport. Recently, it has become increasingly apparent that iron contributes to the development of cancer. In 1959, the first report of malignant tumors induced by iron dextran injection into rats was published (Richmond, 1959). Sarcomas developed in patients injected with iron preparations later confirmed this observation (Greenberg, 1976). According to epidemiological studies from the 1980s, high body iron levels are correlated with a higher risk of cancer (Stevens et al., 1988). As a result of iron overload, the Fenton reaction creates oxidative stress, leading to a specific type of cell death called ferroptosis (Fernández-Mendívil et al., 2021). *In vivo*, iron metabolism and homeostasis are linked with the TME, while ferroptosis plays an important role in tumor immunity (Lu et al., 2021). In a previous study, 18 Ferroptosis-related genes (FRG) were used to divide breast cancer patients into three clusters to improve immunotherapy outcomes, and an immunomicroenvironment and therapeutic response can be prognostically predicted by the FRG signature (Xu et al., 2022). Based on the hallmark gene sets, ATP6AP1 expression was positively associated only with the myogenesis pathway. The AKT signaling pathway is important in myogenesis (Yen et al., 2010), and breast cancer is highly influenced by the AKT signaling pathway. Further research is needed to elucidate the underlying mechanisms. Therefore, our findings suggest that inhibiting APT6AP1 may provide a new and effective method for targeting and interfering with iron metabolism in tumor cells.

A PPI network, including ATP6AP1 and its co-expression genes, was constructed, and the TFs and miRNAs related to ATP6AP1 in breast cancer were identified. In breast cancer, these genes are likely to participate in the regulatory network of ATP6AP1. A solid foundation was laid for future laboratory research using these regulatory networks. Based on the CTD, a drug-gene interaction network was constructed, including ATP6AP1 and related chemical drugs. These drugs targeting ATP6AP1 may inhibit the occurrence and development of tumors by interfering with iron metabolism. Other approaches that hinder tumor growth can also target iron homeostasis. Several reports have shown the antitumor effects of iron chelators such as 3-AP and DFO (Yu et al., 2006). When combined with iron chelators, these drugs targeting ATP6AP1 may exhibit synergistic cytotoxicity against breast cancer cells.

In the present study, we discovered that ATP6AP1 was substantially related to age, ER status, and PR status. ATP6AP1 expression levels might allow us to predict outcomes better than traditional prognostic patterns. In addition, a nomogram with a comprehensive evaluation combining ATP6AP1 with other important clinicopathological parameters was constructed. According to the calibration plot, the actual and anticipated survival probabilities were quite constant. TCGA data were randomly distributed into training and testing sets, and the ROC curves and DCA curves were performed to confirm that the model is valuable for predicting the prognosis (Merath et al., 2019). With our nomogram, patients with breast cancer can obtain personalized scores. In the future, our nomogram could serve as a valuable new prognostic tool for clinicians.

In conclusion, there was a significant increase in ATP6AP1 expression in breast cancer tissues, and greater ATP6AP1 expression was associated with poor prognosis. ATP6AP1 might be an indicator of the inhibition of the immune response to cancer cells and the promotion of iron metabolism for tumor progression. Additional experimental validation is required to demonstrate the biological impact of ATP6AP1. ATP6AP1 may be a new diagnostic, therapeutic, and prognostic target for breast cancer treatment. Our study has certain limitations. First, the sample size was small, and we need an external validation dataset to confirm our conclusions. Second, we collected primary and validation cohorts from the datasets, and intervention details were not included. Third, as the number of healthy subjects in the present investigation was substantially different from that of patients with breast cancer, additional studies are necessary to balance the sample size. Finally, *in vitro* and *in vivo* studies are required to verify our findings.

## Data Availability

The original contributions presented in the study are included in the article/Supplementary Material, further inquiries can be directed to the corresponding author.
