# Supplementary material for: ATP6AP1 is a potential prognostic biomarker and is associated with iron metabolism in breast cancer
Source: Front Genet. 2022 Sep 6;13:958290. doi: 10.3389/fgene.2022.958290 (PMC9486317; doi:10.3389/fgene.2022.958290)
Supplement: Supplementary file 1 [file Table1.DOCX]

GO enrichment analysis

| ONTOLOGY | ID | Description | GeneRatio | BgRatio | pvalue | p.adjust | qvalue |
| --- | --- | --- | --- | --- | --- | --- | --- |
| BP | GO:2000696 | regulation of epithelial cell differentiation involved in kidney development | 5/216 | 19/18670 | 2.02e-06 | 0.006 | 0.006 |
| BP | GO:0035850 | epithelial cell differentiation involved in kidney development | 6/216 | 44/18670 | 1.09e-05 | 0.012 | 0.011 |
| BP | GO:0072182 | regulation of nephron tubule epithelial cell differentiation | 4/216 | 13/18670 | 1.15e-05 | 0.012 | 0.011 |
| BP | GO:1902600 | proton transmembrane transport | 10/216 | 163/18670 | 2.06e-05 | 0.012 | 0.011 |
| BP | GO:0072160 | nephron tubule epithelial cell differentiation | 4/216 | 15/18670 | 2.15e-05 | 0.012 | 0.011 |
| CC | GO:0033176 | proton-transporting V-type ATPase complex | 5/228 | 26/19717 | 1.07e-05 | 0.004 | 0.003 |
| CC | GO:0016469 | proton-transporting two-sector ATPase complex | 5/228 | 51/19717 | 3.01e-04 | 0.038 | 0.035 |
| CC | GO:0005774 | vacuolar membrane | 14/228 | 412/19717 | 3.32e-04 | 0.038 | 0.035 |
| MF | GO:0036442 | proton-exporting ATPase activity | 5/215 | 30/17697 | 2.81e-05 | 0.013 | 0.012 |
| MF | GO:0046961 | proton-transporting ATPase activity, rotational mechanism | 4/215 | 22/17697 | 1.31e-04 | 0.030 | 0.027 |
| MF | GO:0015078 | proton transmembrane transporter activity | 8/215 | 133/17697 | 2.23e-04 | 0.034 | 0.031 |
| MF | GO:0044769 | ATPase activity, coupled to transmembrane movement of ions, rotational mechanism | 4/215 | 36/17697 | 9.20e-04 | 0.075 | 0.068 |
| MF | GO:0016298 | lipase activity | 7/215 | 127/17697 | 9.24e-04 | 0.075 | 0.068 |
