# Supplementary material for: ATP6AP1 is a potential prognostic biomarker and is associated with iron metabolism in breast cancer
Source: Front Genet. 2022 Sep 6;13:958290. doi: 10.3389/fgene.2022.958290 (PMC9486317; doi:10.3389/fgene.2022.958290)
Supplement: Supplementary file 3 [file Table2.DOCX]

KEGG enrichment analysis

| Ontology | ID | Description | GeneRatio | BgRatio | pvalue | p.adjust | qvalue |
| --- | --- | --- | --- | --- | --- | --- | --- |
| KEGG | hsa05120 | Epithelial cell signaling in Helicobacter pylori infection | 8/111 | 70/8076 | 4.60e-06 | 5.54e-04 | 4.69e-04 |
| KEGG | hsa05323 | Rheumatoid arthritis | 9/111 | 93/8076 | 4.64e-06 | 5.54e-04 | 4.69e-04 |
| KEGG | hsa05165 | Human papillomavirus infection | 15/111 | 331/8076 | 4.28e-05 | 0.003 | 0.003 |
| KEGG | hsa05110 | Vibrio cholerae infection | 6/111 | 50/8076 | 5.72e-05 | 0.003 | 0.003 |
| KEGG | hsa00190 | Oxidative phosphorylation | 9/111 | 133/8076 | 8.31e-05 | 0.004 | 0.003 |
| KEGG | hsa04928 | Parathyroid hormone synthesis, secretion and action | 8/111 | 106/8076 | 9.75e-05 | 0.004 | 0.003 |
| KEGG | hsa05146 | Amoebiasis | 7/111 | 102/8076 | 4.84e-04 | 0.017 | 0.014 |
| KEGG | hsa04721 | Synaptic vesicle cycle | 6/111 | 78/8076 | 6.77e-04 | 0.020 | 0.017 |
| KEGG | hsa04072 | Phospholipase D signaling pathway | 8/111 | 148/8076 | 9.46e-04 | 0.025 | 0.021 |
| KEGG | hsa04921 | Oxytocin signaling pathway | 8/111 | 154/8076 | 0.001 | 0.028 | 0.024 |
| KEGG | hsa04014 | Ras signaling pathway | 10/111 | 232/8076 | 0.001 | 0.028 | 0.024 |
| KEGG | hsa00564 | Glycerophospholipid metabolism | 6/111 | 98/8076 | 0.002 | 0.042 | 0.036 |
| KEGG | hsa04010 | MAPK signaling pathway | 11/111 | 294/8076 | 0.002 | 0.042 | 0.036 |
