# Supplementary material for: ATP6AP1 is a potential prognostic biomarker and is associated with iron metabolism in breast cancer
Source: Front Genet. 2022 Sep 6;13:958290. doi: 10.3389/fgene.2022.958290 (PMC9486317; doi:10.3389/fgene.2022.958290)
Supplement: Supplementary file 4 [file Table5.DOCX]

Univariate and multivariate Cox regression analyses

| Characteristics | Total(N) | Univariate analysis | |  | Multivariate analysis | |
| --- | --- | --- | --- | --- | --- | --- |
|  |  | Hazard ratio (95% CI) | P value |  | Hazard ratio (95% CI) | P value |
| T stage | 1079 |  |  |  |  |  |
| T1 | 276 | Reference |  |  |  |  |
| T2 | 629 | 1.332 (0.887-1.999) | 0.166 |  | 0.886 (0.368-2.138) | 0.788 |
| T3&T4 | 174 | 1.953 (1.221-3.123) | **0.005** |  | 1.841 (0.625-5.420) | 0.268 |
| N stage | 1063 |  |  |  |  |  |
| N0 | 514 | Reference |  |  |  |  |
| N1 | 357 | 1.956 (1.329-2.879) | **<0.001** |  | 1.324 (0.614-2.856) | 0.474 |
| N2 | 116 | 2.519 (1.482-4.281) | **<0.001** |  | 1.025 (0.282-3.721) | 0.970 |
| N3 | 76 | 4.188 (2.316-7.574) | **<0.001** |  | 2.446 (0.679-8.812) | 0.171 |
| M stage | 922 |  |  |  |  |  |
| M0 | 902 | Reference |  |  |  |  |
| M1 | 20 | 4.254 (2.468-7.334) | **<0.001** |  | 5.927 (0.966-36.345) | 0.054 |
| Pathologic stage | 1059 |  |  |  |  |  |
| Stage I | 180 | Reference |  |  |  |  |
| Stage II | 619 | 1.697 (0.985-2.922) | 0.057 |  | 0.922 (0.282-3.016) | 0.893 |
| Stage III | 242 | 2.962 (1.664-5.273) | **<0.001** |  | 2.112 (0.366-12.174) | 0.403 |
| Stage IV | 18 | 11.607 (5.569-24.190) | **<0.001** |  |  |  |
| Race | 993 |  |  |  |  |  |
| Asian | 60 | Reference |  |  |  |  |
| Black or African American | 180 | 1.525 (0.463-5.024) | 0.488 |  |  |  |
| White | 753 | 1.325 (0.420-4.186) | 0.631 |  |  |  |
| Histological type | 977 |  |  |  |  |  |
| Infiltrating Ductal Carcinoma | 772 | Reference |  |  |  |  |
| Infiltrating Lobular Carcinoma | 205 | 0.827 (0.526-1.299) | 0.410 |  |  |  |
| PR status | 1029 |  |  |  |  |  |
| Negative | 342 | Reference |  |  |  |  |
| Positive | 687 | 0.732 (0.523-1.024) | 0.068 |  | 0.999 (0.446-2.240) | 0.999 |
| ER status | 1032 |  |  |  |  |  |
| Negative | 240 | Reference |  |  |  |  |
| Positive | 792 | 0.712 (0.495-1.023) | 0.066 |  | 0.421 (0.180-0.983) | **0.046** |
| HER2 status | 715 |  |  |  |  |  |
| Negative | 558 | Reference |  |  |  |  |
| Positive | 157 | 1.593 (0.973-2.609) | 0.064 |  | 1.062 (0.591-1.908) | 0.841 |
| Age | 1082 |  |  |  |  |  |
| <=60 | 601 | Reference |  |  |  |  |
| >60 | 481 | 2.020 (1.465-2.784) | **<0.001** |  | 2.958 (1.759-4.976) | **<0.001** |
| ATP6AP1 | 1082 |  |  |  |  |  |
| Low | 541 | Reference |  |  |  |  |
| High | 541 | 1.417 (1.029-1.951) | **0.033** |  | 1.543 (0.943-2.525) | 0.084 |
