# Supplementary material for: ATP6AP1 is a potential prognostic biomarker and is associated with iron metabolism in breast cancer
Source: Front Genet. 2022 Sep 6;13:958290. doi: 10.3389/fgene.2022.958290 (PMC9486317; doi:10.3389/fgene.2022.958290)
Supplement: Supplementary file 5 [file Table3.DOCX]

| Description | setSize | enrichmentScore | NES |
| --- | --- | --- | --- |
| REACTOME_IRON_UPTAKE_AND_TRANSPORT | 58 | 0.5691 | 2.5054 |
| WP_PROTEASOME_DEGRADATION | 59 | 0.4547 | 2.0117 |
| REACTOME_ROS_AND_RNS_PRODUCTION_IN_PHAGOCYTES | 36 | 0.5099 | 1.9909 |
| KEGG_GLUTATHIONE_METABOLISM | 50 | 0.3664 | 1.5419 |
| KEGG_PYRUVATE_METABOLISM | 39 | 0.3584 | 1.4420 |
| WP_RAS_SIGNALING | 177 | -0.3193 | -1.4379 |
| PID_P53_DOWNSTREAM_PATHWAY | 132 | -0.3709 | -1.6317 |
| WP_HIPPOYAP_SIGNALING_PATHWAY | 23 | -0.5392 | -1.6803 |
| WP_HIPPOMERLIN_SIGNALING_DYSREGULATION | 115 | -0.4072 | -1.7552 |
| WP_MECHANOREGULATION_AND_PATHOLOGY_OF_YAPTAZ_VIA_HIPPO_AND_NONHIPPO_MECHANISMS | 45 | -0.4953 | -1.8024 |
| PID_ECADHERIN_STABILIZATION_PATHWAY | 38 | -0.5662 | -1.9641 |
| WP_INTERACTIONS_BETWEEN_IMMUNE_CELLS_AND_MICRORNAS_IN_TUMOR_MICROENVIRONMENT | 28 | -0.7169 | -2.3309 |

GSEA enrichment analysis
