# Supplementary material for: ATP6AP1 is a potential prognostic biomarker and is associated with iron metabolism in breast cancer
Source: Front Genet. 2022 Sep 6;13:958290. doi: 10.3389/fgene.2022.958290 (PMC9486317; doi:10.3389/fgene.2022.958290)
Supplement: Supplementary file 6 [file Table4.DOCX]

Association between ATP6AP1 expression and clinicopathologic features of patients with breast cancer

| Characteristic | Low expression of ATP6AP1 | High expression of ATP6AP1 | p |
| --- | --- | --- | --- |
| n | 541 | 542 |  |
| T stage, n (%) |  |  | 0.159 |
| T1 | 149 (13.8%) | 128 (11.9%) |  |
| T2 | 310 (28.7%) | 319 (29.5%) |  |
| T3 | 70 (6.5%) | 69 (6.4%) |  |
| T4 | 12 (1.1%) | 23 (2.1%) |  |
| N stage, n (%) |  |  | 0.064 |
| N0 | 282 (26.5%) | 232 (21.8%) |  |
| N1 | 167 (15.7%) | 191 (18%) |  |
| N2 | 56 (5.3%) | 60 (5.6%) |  |
| N3 | 34 (3.2%) | 42 (3.9%) |  |
| M stage, n (%) |  |  | 0.836 |
| M0 | 450 (48.8%) | 452 (49%) |  |
| M1 | 9 (1%) | 11 (1.2%) |  |
| Pathologic stage, n (%) |  |  | 0.460 |
| Stage I | 98 (9.2%) | 83 (7.8%) |  |
| Stage II | 313 (29.5%) | 306 (28.9%) |  |
| Stage III | 113 (10.7%) | 129 (12.2%) |  |
| Stage IV | 8 (0.8%) | 10 (0.9%) |  |
| PR status, n (%) |  |  | < 0.001 |
| Negative | 212 (20.5%) | 130 (12.6%) |  |
| Indeterminate | 2 (0.2%) | 2 (0.2%) |  |
| Positive | 303 (29.3%) | 385 (37.2%) |  |
| ER status, n (%) |  |  | < 0.001 |
| Negative | 169 (16.3%) | 71 (6.9%) |  |
| Indeterminate | 1 (0.1%) | 1 (0.1%) |  |
| Positive | 348 (33.6%) | 445 (43%) |  |
| HER2 status, n (%) |  |  | 0.175 |
| Negative | 291 (40%) | 267 (36.7%) |  |
| Indeterminate | 3 (0.4%) | 9 (1.2%) |  |
| Positive | 82 (11.3%) | 75 (10.3%) |  |
| Race, n (%) |  |  | 0.544 |
| Asian | 26 (2.6%) | 34 (3.4%) |  |
| Black or African American | 93 (9.4%) | 88 (8.9%) |  |
| White | 378 (38%) | 375 (37.7%) |  |
| Age, meidan (IQR) | 55 (47, 65) | 61 (50, 69) | < 0.001 |
